# *Tundrisphaera macrotermitis* sp. nov., a novel member of the family *Isosphaeraceae* isolated from the gut of a fungus-growing termite

**DOI:** 10.1038/s41598-025-20237-w

**Published:** 2025-11-10

**Authors:** Nicolai Kallscheuer, Kim-Loreen Carlstedt, Jonathan Hammer, Tom Haufschild, René Benndorf, Z. Wilhelm de Beer, Michael Poulsen, Christine Beemelmanns, Christian Jogler

**Affiliations:** 1 Department of Microbial Interactions, Institute for Microbiology, Friedrich Schiller University, Jena, Germany; 2 Biochemistry of Microbial Metabolism, Institute of Biochemistry, University of Leipzig, Leipzig, Germany; 3 Department of Biochemistry, Genetics and Microbiology, Forestry and Agricultural Biotechnology Institute, University of Pretoria, Pretoria, South Africa; 4 Section for Ecology and Evolution, Department of Biology, University of Copenhagen, Copenhagen, Denmark; 5 Helmholtz Institute for Pharmaceutical Research Saarland (HIPS), Saarbrücken, Germany; 6 Saarland University, Saarbrücken, Germany; 7 Cluster of Excellence Balance of the Microverse, Friedrich Schiller University, Jena, Germany

**Keywords:** *Planctomycetota*, Soil bacteria, *Macrotermes natalensis*, South Africa, *Tundrisphaera lichenicola*, Computational biology and bioinformatics, Genetics, Microbiology, Molecular biology

## Abstract

A pink-pigmented, neutrophilic and mesophilic strain, TA3^T^, was isolated from the hindgut of a fungus-growing termite of the species *Macrotermes natalensis*. Phylogenetic analysis placed the strain in the family *Isosphaeraceae*, order *Isosphaerales*, class *Planctomycetia*, phylum *Planctomycetota*. The isolate turned out to be an aerobic chemoorganoheterotroph capable of growth under microaerobic conditions. Cells are non-motile, spherical, and either form shapeless aggregates or grow as single cells. The average cell size (length x width) is 2.5 ± 0.3 μm x 2.3 ± 0.2 μm. Cells divide asymmetrically by budding. Optimum pH and temperature for growth are 7.5 (range 6.0–9.0) and 24 °C (range 18–28 °C), respectively. The strain has a genome size of 7.23 Mbp with 69.3% DNA G + C content and it contains four plasmids. Since the genome of the currently known closest relative *Tundrisphaera lichenicola* has not been sequenced, the previously characterized type strain P12^T^ was included for genome sequencing. A comparison based on established phylogenetic markers yielded a 16S rRNA gene sequence similarity of 94.8%, an average nucleotide identity of 78.4% and a digital DNA-DNA hybridization (dDDH) value of 20.3%, suggesting a relationship of the two strains on the level of the same genus. Differences in genome-encoded features, e.g. carbohydrate-active enzymes, secondary metabolite-associated biosynthetic gene clusters and plasmid-located genes were analyzed using comparative genomics. Together with whole genome-based phylogenetic analyses and differences in phenotypic characteristics, the data justifies the delineation of the novel isolate from the sole known species in the genus *Tundrisphaera*. We therefore introduce *Tundrisphaera macrotermitis* sp. nov. that is represented by TA3^T^ (= CECT 30560^T^ = STH00997^T^) as the type strain.

## Introduction

The phylum *Planctomycetota* represents a distinctive group of bacteria known for their unusual cell biology and ecological adaptability^1,2^. Phylum members are recognized for an uncommon cellular architecture including extensive membrane invaginations, condensed DNA and asymmetric cell division by budding^3–6^. Cultivation-dependent and -independent studies have identified members of the two validly described classes *Planctomycetia* and *Phycisphaerae*, and the two provisional classes *Candidatus* Brocadiia and *Candidatus* Uabimicrobiia in a range of habitats. These habitats include freshwater^7,8, ^marine^9–12 ^and terrestrial environments^13,14^. In aquatic ecosystems, higher abundances are typically observed on biotic and abiotic surfaces than in the surrounding water^15,16^. This is in line with the finding that planctomycetes are talented degraders of phototroph-derived polysaccharides^17^, which requires physical contact with micro- and macroscopic phototrophs such as cyanobacteria, seagrasses, macroalgae, etc. ^16^. Consequently, planctomycetes play important roles in global biogeochemical cycles in carbon but also nitrogen transformations; the latter in particular with regard to anaerobic ammonium oxidation (anammox) metabolism performed by members of the class *Ca*. Brocadiia^18^.

Within the well-sudied *Planctomycetia*^2 the family* Isosphaeraceae* (order Isosphaerales) comprises aerobic to facultatively anaerobic, spherical bacteria capable of thriving in both, hot and cold environments^^19–21^. Most *Isosphaeraceae* members have been isolated from peat bogs, lichen-dominated soil and limnic environments^8,22^. Polysaccharide degradation capabilities in the family have been investigated both by cultivation studies and comparative genomics^23–26^.

Planctomycetes have also been found in host-associated environments, such as, the gastrointestinal tracts of insects, with relative abundances of up to 33% ^27–29^, though their functional contributions in these niches remain poorly understood. The gut of the fungus-growing termite species *Macrotermes natalensis* hosts a complex but stable microbial community within which the phylum *Planctomycetota* is among the six most abundant phyla^30^. However, the isolation and characterization of planctomycetes from termite guts also remain sparse, leaving gaps in our understanding of potential adaptations to host-associated lifestyles.

Here, we describe strain TA3^T^ that was isolated from the hindgut of *M. natalensis* after cultivation under aerobic conditions for several months. The isolate belongs to the family *Isosphaeraceae* (phylum *Planctomycetota*), which currently consists of 16 described species in seven genera. The closest related known species, *Tundrisphaera lichenicola*, is constituted by two strains that were isolated from peatland or tundra soil in Russia^22^. Their genomes have not been sequenced yet.

The characterization of strain TA3 is accompanied by genome sequencing of the type strain of *T. lichenicola*. The determination of the genome sequence of the previously isolated strain allowed for more detailed phylogenetic analyses, including whole genome-based markers that, together with differences in phenotypic characteristics, support the delineation of strain TA3 from the other described species in the genus *Tundrisphaera*.

## Materials and methods

### Sampling, strain isolation and sequencing of the 16 S rRNA gene

**Fig. 1:**
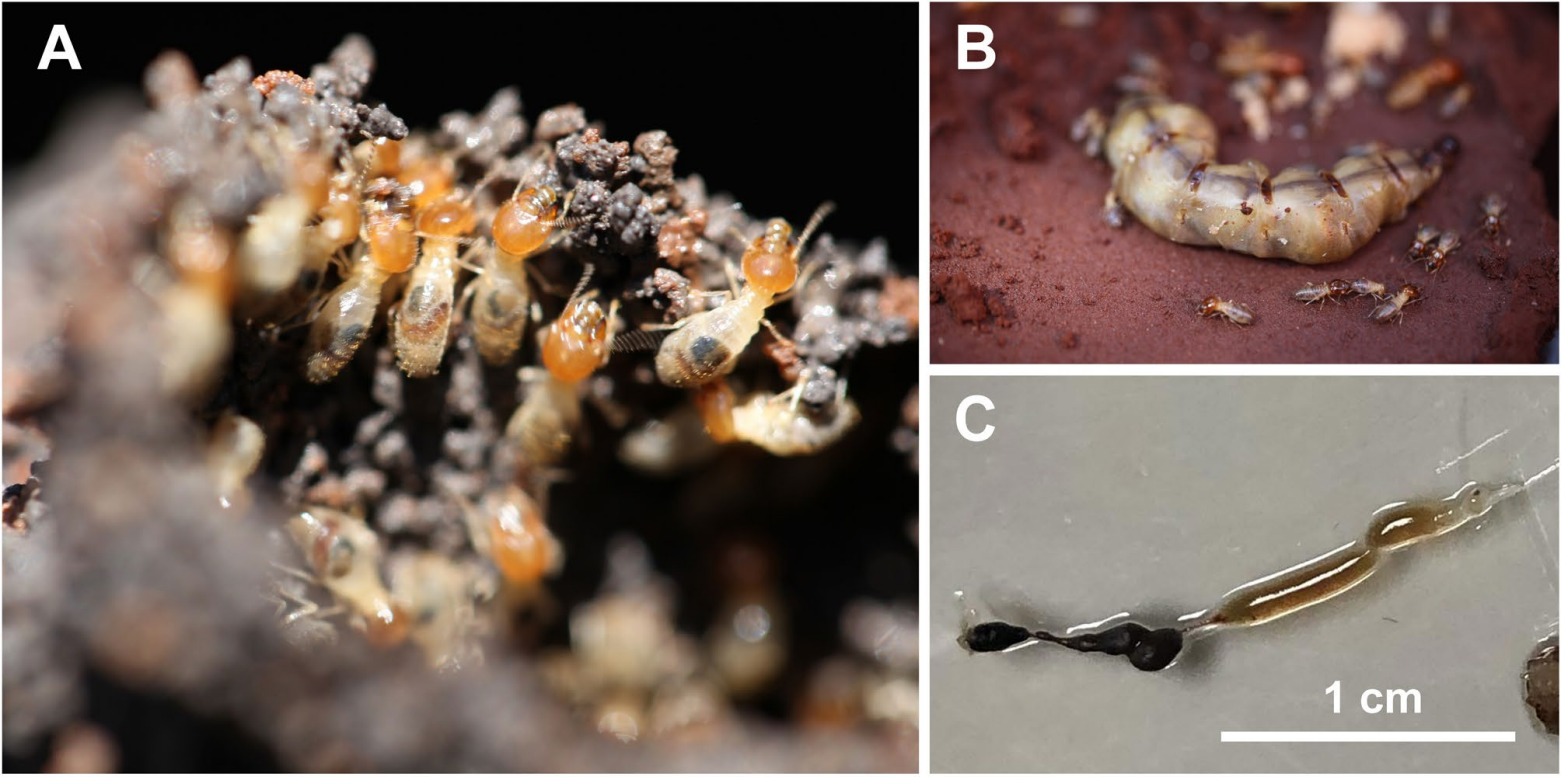
Isolation of strain TA3^T^ from a* Macrotermes natalensis* colony. (**A**) Termite workers use a mixture of soil and saliva to construct walls and mounts that serve to protect the colony. (**B**) Termite workers dedicated to the welfare of the queen, transporting eggs and feeding her. (C) Digestive tract from which strain TA3^T^ was isolated. The hindgut is indicated by the white arrow.

Termites of the species *M. natalensis* were collected from the University of Pretoria Experimental Farm in Pretoria, South Africa in 2016 (latitude − 25.743028, longitude 28.260972) (Fig. 1). Termite specimens were transferred to the laboratory and stored in 50% glycerol. Termites were dissected and split into five parts: crop, foregut, midgut, hindgut, and the remaining termite body. Individual parts were transferred into 1.5 mL reaction tubes with 200 µL of autoclaved water and antifungals (50 µg/mL cycloheximide and 100 µg/mL nystatin). After 24 h of incubation at 4 °C in presence of the antifungals, 10 µL of each sample was transferred to limnic M1 agar supplemented with 200 mg/L ampicillin and 500 mg/L streptomycin prepared as previously described^31^. The plates were incubated under aerobic conditions at 18 °C in plastic boxes. Plates were sealed with Parafilm and incubated with wet papers towels to avoid that plates dry out during longer incubation periods. After five months of incubation, a pinkish colony was visible on the plate inoculated with the hindgut sample. The pinkish colony, designated strain TA3^T^, was re-streaked on fresh plates and was maintained in liquid limnic M1 medium without antibiotics. The 16S rRNA gene of strain TA3^T^ was amplified by PCR, purified based on a standardized workflow^32^ and sequenced at Macrogen Europe (Amsterdam, The Netherlands).

### Physiological analyses

For determination of the temperature optimum for growth, 100 µL supernatant of an exponentially growing culture were plated and plates were incubated in triplicates at temperatures ranging from 4 to 37 °C. Plates were checked daily, and growth was evaluated by the time required until visible colonies were formed. The temperature at which colonies appeared the earliest was considered the temperature optimum for growth. The pH optimum for growth was determined in liquid cultures of limnic M3 medium (same recipe as limnic M1 medium but with amounts of peptone and yeast extract increased to 1 g/L each). As buffering agents, the medium contained either 100 mM of 2-(*N*-morpholino)ethanesulfonic acid (MES) for pH 5.0 and 6.0, 4-(2-hydroxyethyl)-1-piperazineethanesulfonic acid (HEPES) for pH 7.0 and 8.0 or *N*-cyclohexyl-2-aminoethanesulfonic acid (CHES) for pH 9.0 and 10.0. Growth was evaluated by measuring the optical density at 600 nm (OD_600_). Growth under anoxic conditions was examined using solidified and liquid limnic M3 medium. The medium was anaerobized by flushing it with nitrogen gas for two minutes until oxygen concentrations below 1 µmol/L were achieved. Plates were then transferred to an anaerobic jar (nox-18, SY-LAB, Neupurkersdorf, Austria). Plates and sealed serum bottles were incubated at 24 °C.

### Microscopy and cell size determination

For cell size determination and fluorescence microscopy, a Nikon Eclipse Ti2 inverse microscope with two different set-ups was used as described earlier^33^. To analyze cell size, cells of strain TA3^T^ were handled and images were analyzed as previously described^31^. Briefly, cells grown in limnic M3 medium were mounted on a 1% (w/v) agarose cushion dissolved in the same medium. Images were loaded into FIJI^34^, all three RGB-channels were combined and tiff files were transferred to BacStalk^35^. Segmentation settings were adjusted to 25 and 15 pixels for cell size and minimum cell size, respectively. Segmented cells (three replicates with 150 cells each) were checked manually for detection errors. The data was transferred to SuperPlotsOfData^36^. For visualization purposes, brightness and contrast were adjusted manually. For fluorescence microscopy, 500 µL of a culture was stained with 3 µL 4′,6-diamidin-2-phenylindole (DAPI) and 1 µL Synaptored for 30 min. Cells were washed 3 times with limnic M3 medium. After immobilization on a 1% (w/v) agarose pad dissolved in the same medium, cells were imaged with a DAPI filter (Semrock; DAPI-1160B) and a Texas Red longpass filter (AHF; 560/40x, 600 DC, 610 LP). Images were transferred to FIJI (if necessary, images were merged with the *in-FIJI* function) and brightness and contrast were adjusted manually to enhance visibility.

### Genomic DNA isolation, genome sequencing, annotation and analysis

Isolation of genomic DNA as well as genome sequencing, assembly and polishing of strain TA3^T^ were performed as previously described^31^. The genome was assembled *de novo* from Oxford Nanopore long reads and polished with Illumina short reads. Illumina sequencing was performed by Eurofins Genomics (Ebersberg, Germany). *T. lichenicola* P12^T^ was ordered from the Belgian culture collection BCCM/LMG (deposition number LMG 29571) and processed in the same way as strain TA3^T^. The final genome sequences were checked for completeness using BUSCO v5.8.2. Coding density and DNA G + C content were analyzed with checkM v1.2.3. After annotation with prokka v1.14.5, the chromosome was rotated to the start codon of the replication initiator protein-encoding gene *dnaA* and was re-annotated with PGAP version 2025-05-06.build7983. Plasmids were rotated to the start codon of the chromosome partitioning ATPase-encoding gene *parA* when such a protein was annotated.

### Nucleotide sequence accession numbers

The 16S rRNA gene sequences of strain TA3^T^ and *T. lichenicola* P12^T^ are available from the NCBI GenBank database under the accession numbers PV747909 and PV748000, respectively. Genome sequence information for strain TA3^T^ was deposited under the accession numbers CP195100 (chromosome), CP195101 (pTA3_1), CP195102 (pTA3_2), CP195103 (pTA3_3) and CP195104 (pTA3_4). Genome sequence information for *T. lichenicola* P12^T^ was deposited under the accession numbers CP195106 (chromosome), CP195107 (pP12_1) and CP195108 (pP12_2).

### Phylogenetic and genome-based analyses

The 16S rRNA gene sequence of strain TA3^T^ was extracted from the prokka-annotated genome and was used for the identification of the current closest relatives using NCBI BLAST^37^. A maximum-likelihood 16S rRNA gene sequence-based phylogeny was computed for the novel strain and the described type strains of all species in the current phylum *Planctomycetota* (as of June 2025). Three sequences from strains outside of the phylum *Planctomycetota*, but part of the *Planctomycetota-Verrucomicrobiota-Chlamydiota* (PVC) superphylum, namely *Opitutus terrae* (NCBI acc. no. AJ229235), *Kiritimatiella glycovorans* (acc. no. NR_146840) and *Lentisphaera araneosa* (accession number NR_027571), served as outgroup. The alignment of the 16S rRNA gene sequences was performed with ClustalW^38^ and FastTree v2.2 was used for tree reconstruction with 1000 bootstrap replications^39^. A multi-locus sequence analysis (MLSA)-based phylogeny was performed using autoMLST with 500 bootstrap replicates^40^. The analysis included the genomes of all described members of the family *Isospheraceae* and genomes of *Rhodopirellula baltica* SH1^T^ (GenBank acc. no. BX119912.1), *Pirellula staleyi* DSM 6068^T^ (acc. no. CP001848.1) and *Blastopirellula marina* DSM 3645^T^ (acc. no. GCA_000153105.1) (all belonging to the family *Pirellulaceae*) served as outgroup. Phylogenetic trees were visualized with iTOL v6 ^41^. The 16S rRNA gene sequence similarity matrix was obtained with TaxonDC^42^ based on the ClustalW alignment that was also used for the construction of the phylogenetic tree. Average amino acid identities (AAI) and average nucleotide identities (ANI) were calculated using respective scripts of the enveomics collection^43^. Additional phylogenetic markers, i.e. *rpoB* sequence similarity and percentage of conserved proteins (POCP), were calculated as described^44,45^. Digital DNA-DNA hybridization (dDDH) values were calculated with the Genome-to-Genome Distance Calculator of the Type Strain Genome Server (TYGS) (https://ggdc.dsmz.de). The pangenome of selected strains was generated with anvi’o v.8 ^46^. Biosynthetic gene clusters (BGCs) were predicted with antiSMASH v.8.0 ^47^ and carbohydrate-active enzymes (CAZymes) with dbCAN3^48^. The presence of phage genes was analyzed with PHASTEST^49^. The clustering of protein sequences encoded by plasmid-located genes was performed with CLANS from the MPI Bioinformatics Toolkit^50^.

## Results and discussion

### Phylogenetic inference

**Fig. 2:**
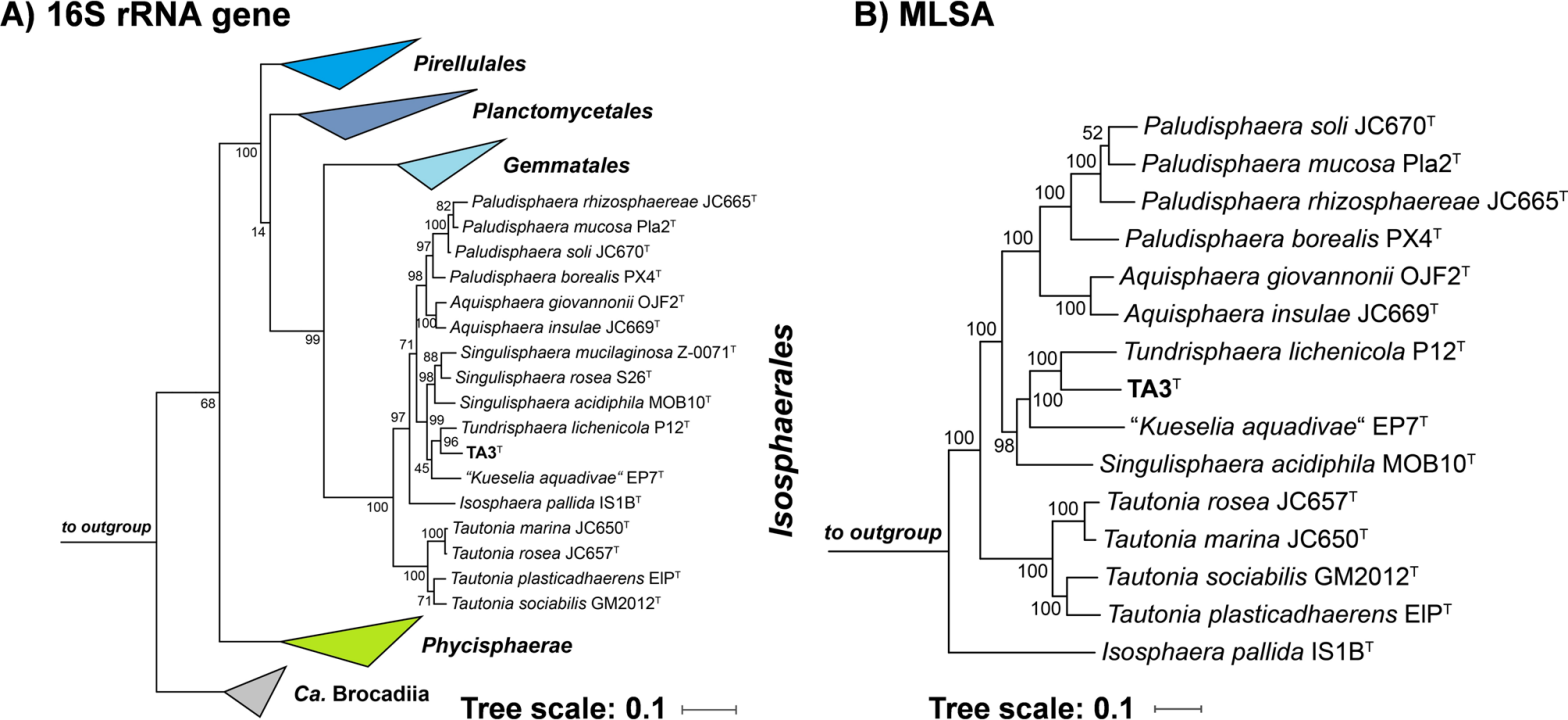
Phylogenetic placement. (**A**) Maximum likelihood phylogenetic tree based on 16S rRNA gene sequences showing the phylogenetic relationship of strain TA3^T^ and the characterized members in the order *Isosphaerales*. FastTree was used for tree reconstruction with 1000 bootstrap replications (given at the nodes, in %) and *Opitutus terrae* (NCBI acc. no. AJ229235), *Kiritimatiella glycovorans* (acc. no. NR_146840) and *Lentisphaera araneosa* (acc. no. NR_027571), served as outgroup. Bar, 0.1 nucleotide substitutions per position. (**B**) Multi-locus sequence analysis (MLSA)-based phylogenetic tree based on the characterized members in the order *Isosphaerales*. The tree was computed based on a set of at least 30 single-copy gene-encoded proteins in a maximum likelihood approach with 500 bootstrap replications using the tool autoMLST (see Materials and Methods section for details). Bootstrap values are given at the nodes. Bar, 0.1 amino acid substitution per position. Phylogenetic trees were visualized with iTOL v6.

**Fig. 3:**
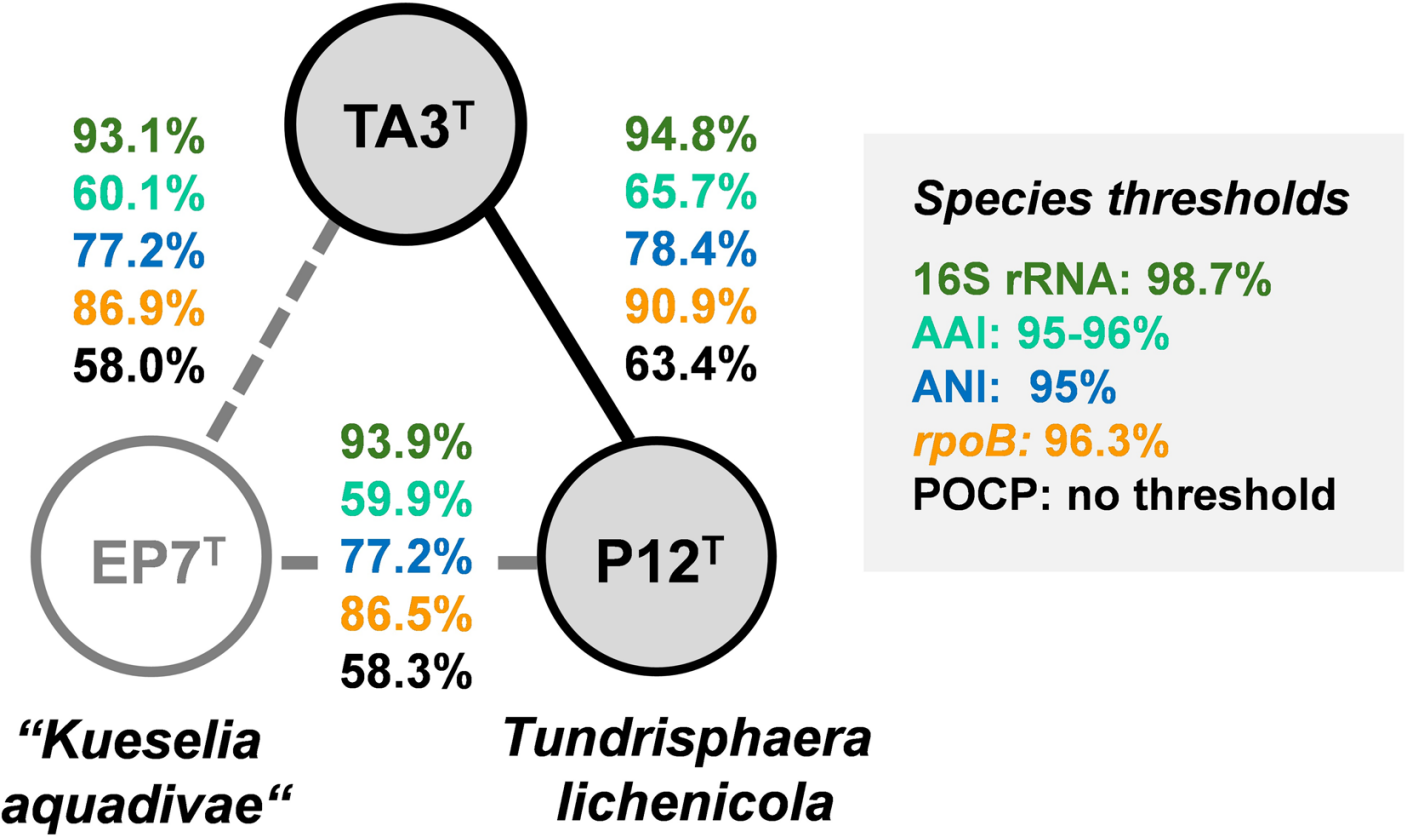
Comparison of phylogenetic markers for species delineation. Markers used: 16S rRNA gene sequence identity (16S rRNA), average amino acid identity (AAI), average nucleotide identity (ANI), sequence similarity of a partial sequence of the *rpoB* gene *(rpoB)*, percentage of conserved proteins (POCP), digital DNA-DNA hybridization (dDDH). Grey-filled circles and full lines indicate a relationship on the genus level.

To estimate the phylogenetic position of the novel strain TA3^T^, its 1508 bp 16S rRNA gene sequence was compared against NCBI’s core nucleotide database using blastn. The analysis yielded highest similarities in the range of 93–95% to two members of the family *Isosphaeraceae*, *T. lichenicola* P12^T^ and the recently described strain “*Kueselia aquadivae*” EP7^T^ ^22,51^. Since the genome of *T. lichenicola* P12^T^ had not been sequenced, the strain was ordered from the Belgian collection BCCM/LMG (deposition number LMG 29571), and its genome sequence was determined along with that of the novel isolate. This allowed for more accurate phylogenetic analyses based on genome-based markers including AAI, ANI, POCP and sequence similarity of a ca. 1300 bp partial sequence of the *rpoB* gene encoding the β-subunit of RNA polymerase. Maximum-likelihood phylogenetic trees based on 16S rRNA gene sequences and MLSA (Fig. 2) substantiated the suspected clustering in proximity to the two mentioned relatives, identifying *T. lichenicola* P12^T^ as the currently closest characterized relative of strain TA3^T^. The exact sequence similarity of 94.8% based on the full-length 16S rRNA genes of strains P12^T^ and TA3^T^ extracted from the genome fell above the accepted genus threshold of 94.5%, but below the species threshold of 98.7% ^52^. The inference that strain TA3^T^ is a novel species in the genus *Tundrisphaera* was supported by the analysis of whole genome-based phylogenetic markers (Fig. 3). The partial *rpoB* sequence similarity of 96.3% obtained during comparison of strains TA3^T^ and P12^T^ is the exact value of the proposed species threshold^44^, whereas values for ANI and AAI were well below the accepted threshold for species delineation^53,54^. While POCP values are typically only used for the delineation of genera (genus threshold 50%) ^45^, the four phylogenetic markers with available species thresholds (16S rRNA gene sequence similarity, ANI, AAI and partial *rpoB* sequence similarity) support the position of TA3^T^ as member of a novel species.

### Comparison of genomic features

The genome of strain TA3^T^ has a size of 7.23 Mbp. It comprises the chromosome and four plasmids. The presence of one to five plasmids is a common feature of the order *Isosphaerales* in comparison to the other orders in the class *Planctomycetia* in which plasmid occurrence in characterized strains is sporadic^23,55^. With 7.80 Mbp, the genome of *T. lichenicola* P12^T^ is slightly larger, but the strain only contains two plasmids. Both genomes have high DNA G + C content that differs by nearly 5 percentage points (Table 1). Strain TA3^T^ has five copies each of 5S, 16S and 23S rRNA genes that are arranged in five *rrn* operons (16S–23S-5S rRNA genes) whereas *T. lichenicola* P12^T^ has four. While the organization of rRNA genes in operons is common in most bacteria, many planctomycetotal species have “unlinked” rRNA genes^56,57^. The numbers of tRNAs, coding sequences per Mbp and coding densities of the three genomes are comparable. Relative numbers of 26–28% of hypothetical protein-encoding genes are in the expected range for planctomycetotal genomes which are among the bacterial genomes with the highest numbers of hypothetical proteins^58^.

**Table 1:** Comparison of phenotypic features.

Characteristics	TA3^T^	*Tundrisphaera lichenicola* P12^T^	“*Kueselia* *aquadivae*” EP7^T^
Sampling Information			
Location	Experimental termite farm, Pretoria (South Africa)	Nadym Region, Yamalo-Nenets Autonomous Okrug (Russia)	Hainich National Park, Thuringia (Germany)
Sampled Material	Hindgut of *Macrotermes natalensis*	Oxic Layer of Peat	Percolate of Drainage Collector
Phenotypic features			
Pigmentation	pink	pink	light pink
Cell Shape	spherical	spherical	spherical
Size (Length x width) (µm)	2.5 × 2.3	2.2-3.0	2.4 × 2.2
Cell Division Mode	asymmetric (polar) cell division (budding)	asymmetric (polar) cell division (budding)	asymmetric (polar) cell division (budding)
Temperature Range (optimum) (°C)	18–28 (24)	4–28 (15–22)	10–25 (18–21)
pH Range (optimum)	6.0–9.0 (7.5)	4.5–6.8 (5.5–6.0)	6.0–9.0 (7.5)
Relation to Oxygen	aerobic, growth under microaerobic conditions	strictly aerobic	facultatively anaerobic
Motility	non-motile	non-motile	non-motile
Stalks	not observed	Holdfast-like appendages	not observed
Aggregates	short chains, or shapeless aggregates	short chains	small, microscopic aggregates

### Pangenome reconstruction, analysis of secondary metabolite-associated biosynthetic gene clusters and carbohydrate-active enzymes

**Fig. 4:**
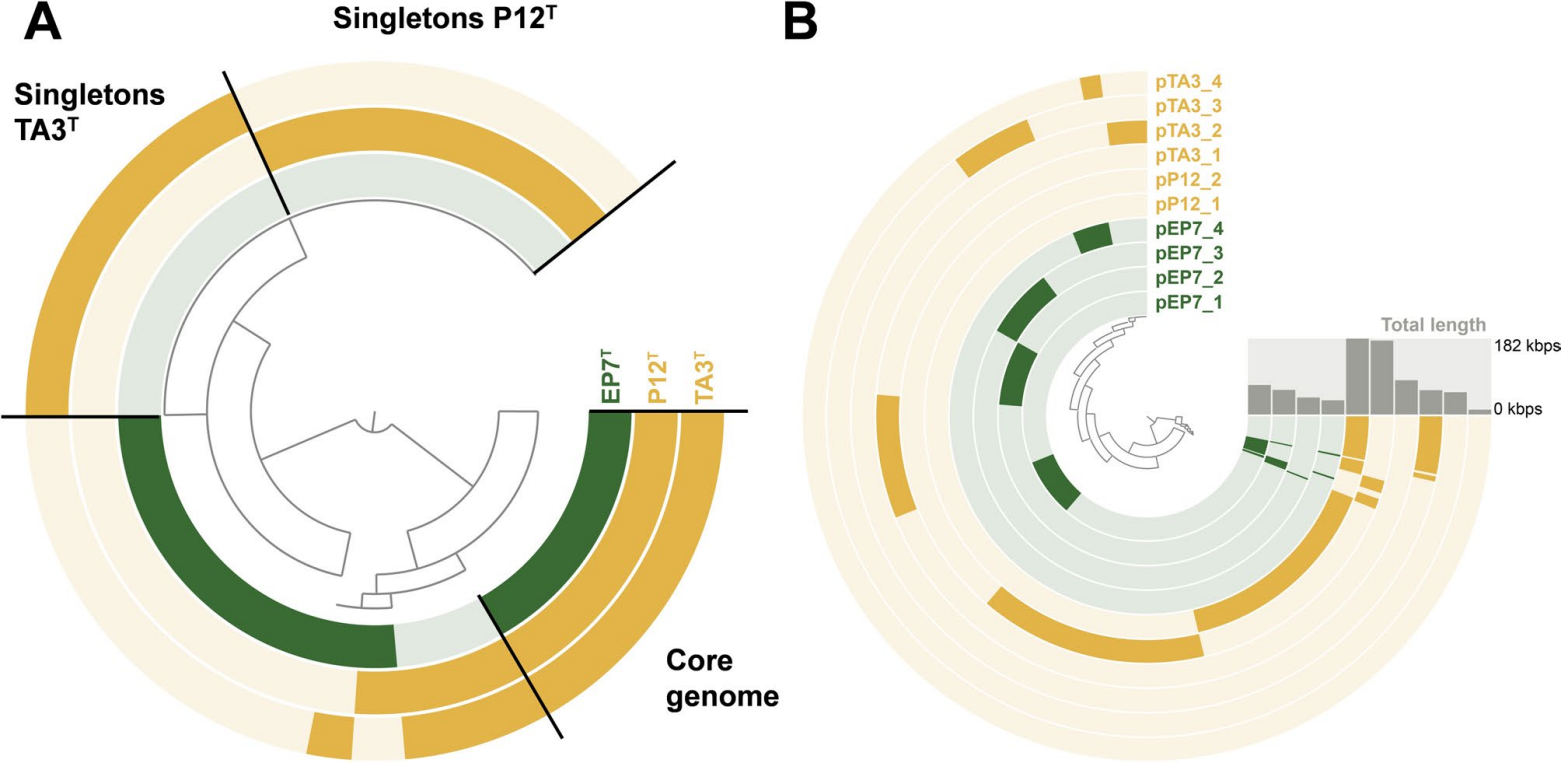
Pangenome and ‘panplasmidome’ analyses. Each open circle represents the pangenome (**A**) or ‘panplasmidome’ (**B**) of all genomes or plasmids, with darker shading indicating the presence of a given gene in the corresponding genome or plasmid. A) The core genome and singletons of the two *Tundrisphaera* species are highlighted. B) ‘Total length’ denotes the combined length of the analyzed plasmids, expressed in kilobase pairs (kbps).

The constructed pangenome based on the genomes of “*K. aquadivae*” EP7^T^ and the two sequenced *Tundrisphaera* spp. consisted of 11,030 clusters, of which 2,078 were present in all three genomes (core genome). The analysis yielded 2,310 singleton genes for strain TA3^T^ and 2,544 for *T. lichenicola* P12^T^ (Fig. 4A). In silico mining of planctomycetotal genomes using antiSMASH is typically performed to get an overview on secondary metabolite biosynthetic capabilities of individual strains and to identify potential talented producers of novel bioactive compounds^4,59,60^. As was also the case for the three strains compared here (Table 2), antiSMASH yielded around one BGC per Mbp for planctomycetotal genomes. The analysis yielded a conserved set of four terpenoid biosynthesis-related BGCs, two type I and one type III polyketide synthase clusters across all three genomes and 1–2 additional strain/species-specific clusters. The latter were predicted to be involved in the biosynthesis of acyl amino acids or ribosomally and non-ribosomally synthesized peptides. AntiSMASH predicted an additional non-ribosomal peptide synthetase-encoding gene in strain TA3^T^ that was absent in the other two genomes.

**Table 2:** Comparison of genomic and genome-encoded features.

Characteristics	TA3^T^	*Tundrisphaera lichenicola* P12^T^	“*Kueselia* *aquadivae*” EP7^T^
Genomic features			
Genome size (bp)	7,233,870	7,799,449	7,199,273
Plasmids	4	2	4
DNA G + C (%)	69.3	64.7	66.7
Genes	5,862	6,200	5,685
Genes/Mbp	810	795	790
Protein-coding genes	5,750	6,057	5,563
Protein-coding genes/Mbp	795	777	773
Hypothetical proteins*	1,540	1,553	1,534
Hypothetical proteins (%)	26.8	25.6	27.6
Coding density (%)	88.1	86.8	86.3
rRNA genes (5S–16S-23S)	5,5,5	4,4,4	4,4,4
tRNA genes	64	51	58
Secondary metabolite-associated biosynthetic gene clusters			
Terpene	4	4	4
Type I polyketide synthase	2	2	3
Type III polyketide synthase	1	1	1
Ribosomally synthesized modified peptide	2	1	0
Non-ribosomal peptide synthetase	1	0	0
Acyl amino acid	0	2	0
BGCs per Mbp	1.4	1.3	1.1
Carbohydrate-active enzymes			
Glycoside hydrolases (GH)	58	50	55
Glycosyltransferases (GT)	90	99	85
Polysaccharide lyases (PL)	3	0	3
Carbohydrate esterases (CE)	19	24	22
Carbohydrate-bind. modules (CBM)	16	23	12
Auxiliary activities (AA)	4	5	2
CAZyme genes	190	201	179
CAZyme genes per Mbp	26	26	25

*Based on the PGAP-annotated genomes

The number of putative CAZyme-encoding genes identified in the three genomes fell in the range of 180–200 with nearly identical values of 25–26 CAZyme genes per Mbp. The sampling spots from which strains TA3^T^ and P12^T^ were isolated differ in several properties, including temperature, humidity, and host association. Hence, we checked whether habitat-specific requirements for the degradation of carbohydrates were reflected in differences in their CAZyme portfolios. However, this revealed only minor differences for the presence/absence of the different subfamilies. In a comparison of strains P12^T^ and TA3^T^, eleven subfamilies were specific for P12^T^ (AA1, CBM47, CBM57, CBM66, CBM67, CE4, GH1, GH5, GH51, GH141, GT1) while tnine subfamilies were specific for TA3^T^ (CBM2, GH24, GH26, GH76, GH123, GH140, GT28, PL10, PL11). Striking was the lack of putative polysaccharide lyases (PLs) in *T. lichenicola* P12^T^ and the presence of different PL subfamilies with partly overlapping specificities in the other two strains (PL10 and PL11 in strain TA3^T^ and PL9 and PL42 in “*K. aquadivae*” EP7^T^). PL9 and PL11 are presumably involved in the degradation of rhamnogalacturonan, PL10 in the degradation of pectate and PL42 of L-rhamnose-α-1,4-D-glucuronate.

Interestingly, none of the TA3^T^-specific CAZyme subfamilies was previously found to be termite specific^61^. This finding along with the large genome of strain TA3^T^ questions if this strain is indeed among the stable symbionts within the termite gut. Planctomycetes are rarely isolated from animals during cultivation attempts; with jellyfish^62^, sponges^63–65^ and - as described in this study - the termite gut as the only examples. In all cases, the interaction of the planctomycetal strains with their putative host remains enigmatic.

### Comparison of plasmid sequences and identification of conserved plasmid genes

All characterized members of the family *Isosphaeraceae* have at least one and maximum five plasmids; however, the physiological role of maintenance of (several) extrachromosomal elements in the family remains enigmatic. The previously sequenced “*K. aquadivae*” EP7^T^ has four plasmids (with lengths 70, 58, 41 and 34 kbps), *T. lichenicola* P12^T^ has two (182 and 177 kbps) and strain TA3^T^ has four (81, 57, 52 and 11 kbps). Since core elements for plasmid replication and distribution in the family have not been identified, we used a “panplasmidome” approach to search for conserved features (Fig. 4B). No conserved genes were present in all plasmids and only two conserved plasmid-located genes were present in all three strains. These genes code for a putative nucleoside-diphosphate-sugar epimerase (WcaG) and a UDP-*N*-acetyl-D-mannosamine dehydrogenase (WecC). WcaG influences cell surface properties, virulence and extracellular enzyme production in the gamma-proteobacterium *Pectobacterium carotovorum*^66^. WecC is involved in the biosynthesis of enterobacterial common antigen, a surface polysaccharide specific for members of the family *Enterobacteriaceae* (class *Gammaproteobacteria*)^67^. Genes encoding putative ParA (ATPase) and ParB (Chromosome-partitioning protein) proteins of the ParABS system for plasmid partitioning were also identified. ParA-encoding genes were annotated on all plasmids except pTA3_4 (the smallest plasmid in the analysis) although in nine different gene clusters generated during the automated pangenome construction by anvi’o (indicating low sequence similarities). Putative ParB-encoding genes were only present on the two largest plasmids each in all three strains (i.e. absent in pEP7_3, pEP7_4, pTA3_3, pTA3_4) and fell in six clusters generated by anvi’o. PHASTEST did not yield any plasmid-located phage genes in either of the 10 analyzed plasmids. The results of the plasmidome analysis could be substantiated by clustering of the protein sequences of plasmid-encoded genes using CLANS (Clustering based on all-against-all BLAST + similarities). Apart from the above-mentioned proteins, the plasmids were rich in genes encoding putative serine/threonine and histidine protein kinases, tyrosine recombinases and proteins annotated as inositol dehydrogenases or inositol glycosyltransferases. As observed previously, plasmids in the family are heterogeneous^55^ and apart from the predicted ParA and ParB proteins that are likely involved in plasmid partitioning, the function of plasmid gene-encoded proteins remains to be elucidated.

### Phenotypic characterization

On agar plates, strain TA3^T^ forms pink-pigmented, smooth and round colonies. When cultivated in larger volumes in limnic M3 medium in stirred bioreactors, the culture has a more orange to reddish color. With shaking, the strain did not form macroscopic aggregates and reached a final OD_600_ of 1.9-2.0 from a starting OD_600_ of 0.05. In limnic M3 medium, the strain grew over a temperature range of 18–28 °C and a pH range of 6.0–9.0. Under optimal conditions (pH 7.5, 24 °C), the strain reached a maximal growth rate of 0.026 h^− 1^, corresponding to a division time of ca. 27 h. The growth was slightly faster than that of *T. lichenicola* P12^T^ at 22°C (generation time 35 h)^22^. Strain TA3^T^ prefers slightly higher temperatures compared to its close relatives but all three strains failed to grow at temperatures > 28 °C (Table 1). The neutrophilic lifestyle of strain TA3^T^ is similar to that of “*K. aquadivae*” EP7^T^ but different from *T. lichenicola* P12^T^ that prefers slightly acidic conditions. In contrast to the strictly aerobic *T. lichenicola* P12^T^, strain TA3^T^ grew under oxygen-limited conditions. The strain could be passaged three times under anaerobic conditions in serum bottles, before turbidity did not increase anymore. Since the strain lacks genes encoding common enzymes involved in anaerobic respiration, e.g. nitrate, nitrite or sulfate reductase and formate dehydrogenase, it is likely that the observed growth under microaerobic conditions involves fermentation. The capability of the strain to grow under such conditions is in line with the finding that the hindgut of termites is typically a low-oxygen environment in which some oxygen can penetrate through diffusion from the termite tracheal system or via ingested materials.

**Fig. 5:**
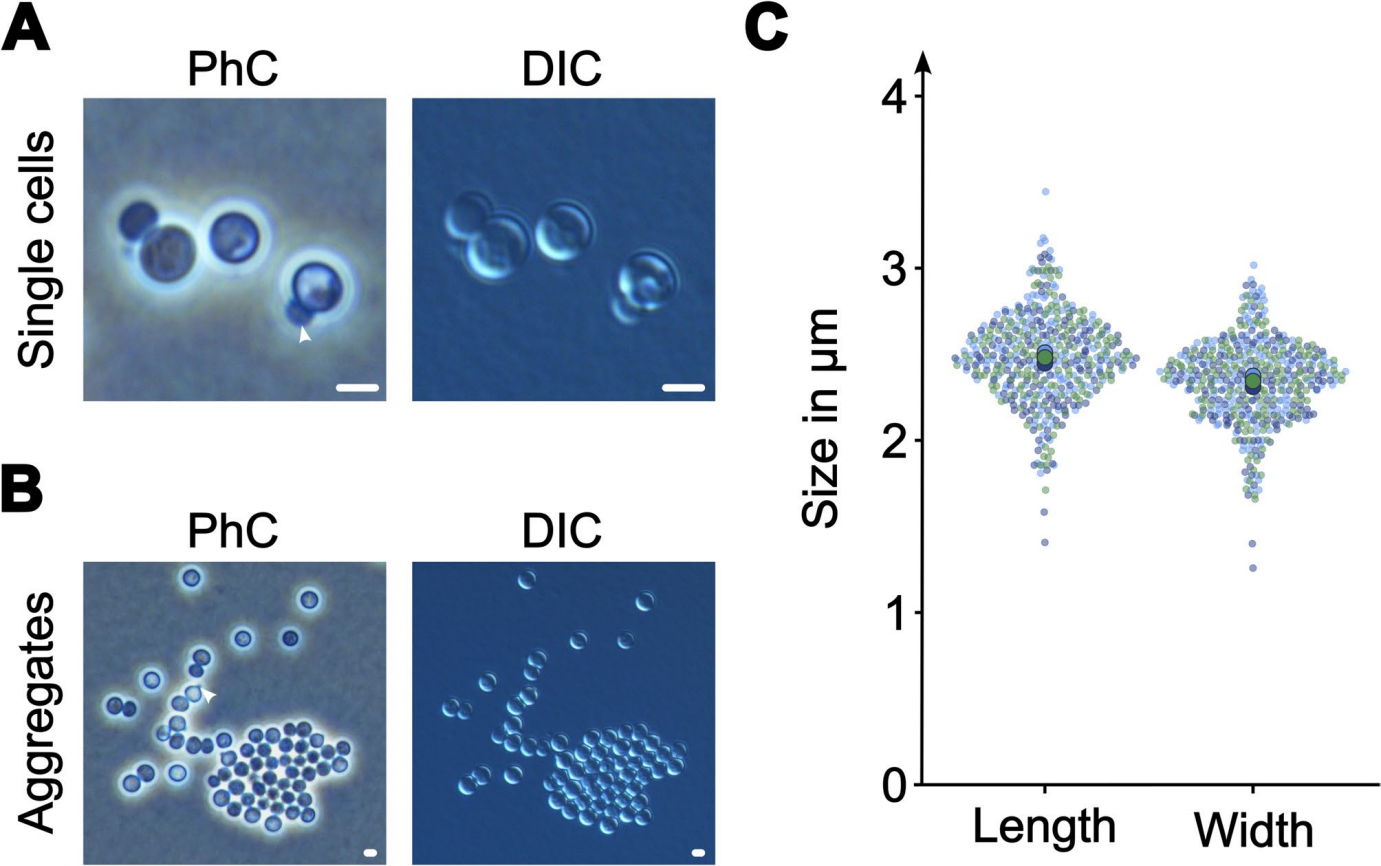
Cell morphology and cell size of strain TA3^T^. (**A**) Microscopic images of cells of strain TA3^T^ captured with phase contrast (PhC) and differential interference contrast (DIC). (**B**) PhC and DIC microscopic image of cell aggregates as well as of individual cells. Dividing cells are indicated by an arrowhead pointing on daughter cells; scale bars are 2 μm. (**C**) Cell sizes determined in three replicates are indicated by three different colors; larger circles indicate the mean values of each replicate. The cell size is 2.5 ± 0.3 × 2.3 ± 0.2 μm (mean ± standard deviation), indicating spherical/oval cell shape.

Cells of strain TA3^T^ are spherical (Fig. 5A), occasionally oval and can form microscopic shapeless aggregates (Fig. 5B). Individual cells have a mean cell length and mean cell width of 2.5 ± 0.3 μm and 2.3 ± 0.2 μm (Fig. 5C), respectively. *T. lichenicola* P12^T^ and “*K. aquadivae”* EP7^T^ have a similar morphology, can also form aggregates, and have similar cell sizes^22,51^. Cells of strain TA3^T^ divide asymmetrically via “budding” where the daughter cell emerges as tiny round structure on one side of the cell, grows and eventually pinches off (Fig. 5); strains P12^T^ and EP7^T^ divide via the same mechanism. In other members of the phylum *Planctomycetota* the division can be further classified based on the localization/orientation of the division plane (polar or lateral)^4^. Such an orientation appears to be absent in spherical cells and the division mechanism/division plane placement mechanism cannot be categorized any further yet.

**Fig. 6:**
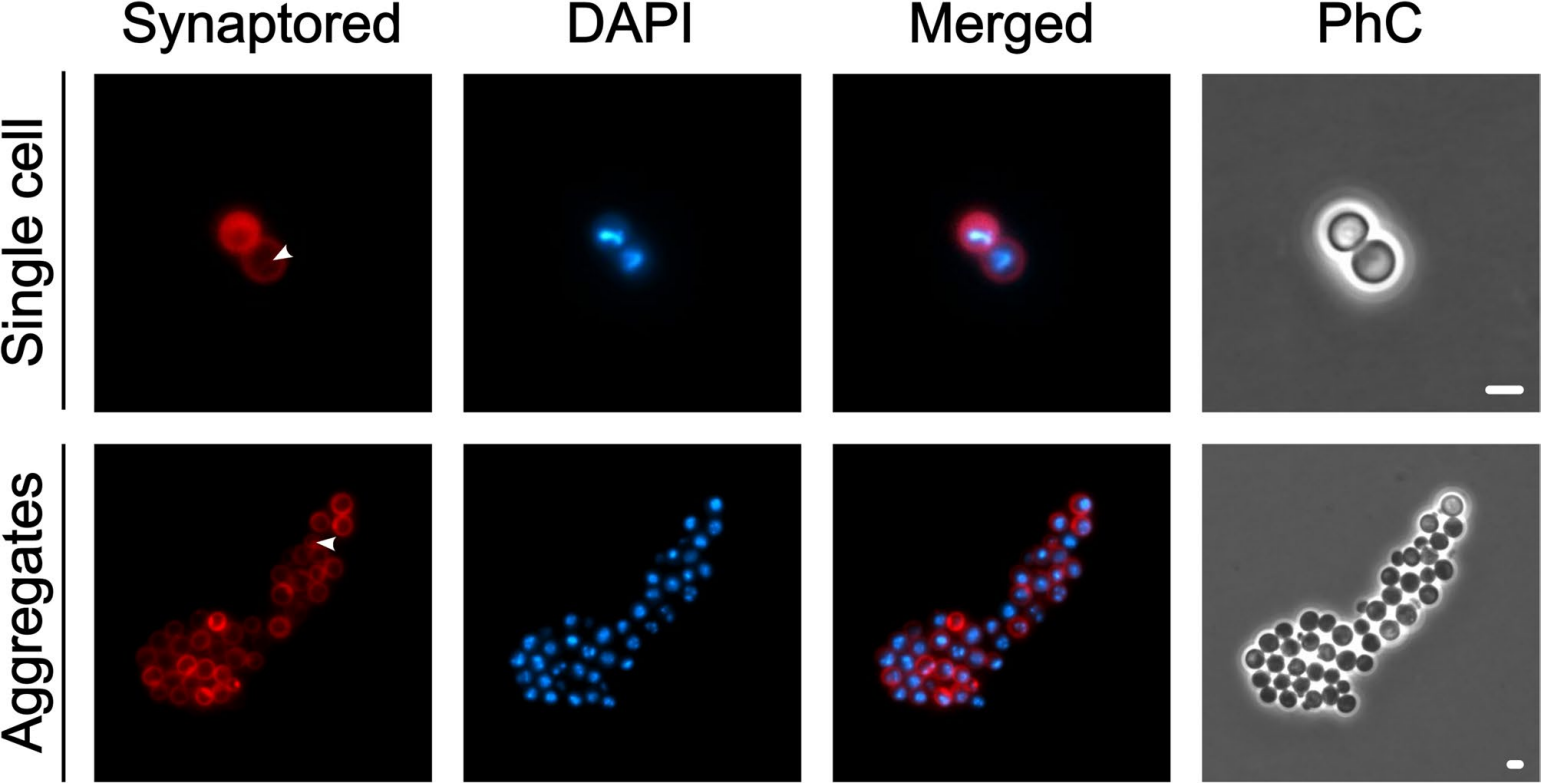
Strain TA3^T^ displays invaginations of the cytoplasmic membrane and a condensed nucleoid. Individual cells and cells in aggregates display cytoplasmic membrane invaginations (stained with Synaptored, arrowheads) and therefore possess an enlarged periplasmic space. Cells possess a condensed nucleoid (stained with DAPI), occasionally cells display multiple smaller DAPI spots. PhC = Phase contrast, scale bars are 2 μm.

Two additional typical traits of planctomycetes include invaginations of the cytoplasmic membrane giving room to an enlarged periplasmic space and a condensed nucleoid^3^. To investigate these characteristics in strain TA3^T^, cells were treated with Synaptored and DAPI, which stain membranes and DNA, respectively. Like for the model strain *Planctopirus limnophila* and “*K. aquadivae”* EP7^T^, cells of strain TA3^T^ displayed invaginations in the cytoplasmic membrane which occasionally spanned through the cytoplasm of the cells. Additionally, (multiple) intense DAPI spots in one cell could be observed pointing towards a condensed nucleoid as present in other planctomycetes (Fig. 6).

## Conclusion

Strain TA3^T^ shows clear distinction from its closest relatives *T. lichenicola* P12^T^ *and* “*K. aquadivae*” EP7^T^ for both, genome-encoded/phylogenetic and phenotypic characteristics. The data supports a position for the novel strain within the genus *Tundrisphaera* but delineated from the sole species *T. lichenicola*. Hence, we propose a novel species, for which we introduce the name *Tundrisphaera macrotermitis* sp. nov.

### Description of* Tundrisphaera macrotermitis* sp. nov.

*Tundrisphaera macrotermitis* (ma.cro.ter’mi.tis. N.L. gen. masc. n. *macrotermitis*, of the termite *Macrotermes*, from which the organism was isolated).

Aerobic chemoorganoheterotroph capable of growth under microaerobic conditions. Colonies are pink-pigmented. Cells are non-motile, spherical, and form shapeless aggregates or grow as single cells. The average cell size is 2.5 ± 0.3 μm and 2.3 ± 0.2 μm (length x width). Cells divide asymmetrically by budding. Optimum pH and temperature for growth are 7.5 (range 6.0–9.0) and 24 °C (range 18–28 °C), respectively. The type strain is TA3^T^ (= CECT 30560^T^ = STH00997^T^; the STH number refers to the Jena Microbial Resource Collection JMRC) and was isolated from the hindgut of a termite of the species *Macrotermes natalensis* sampled in Pretoria, South Africa. The type strain has a genome size of 7.23 Mbp (chromosome and four plasmids) and a DNA G + C content of 69.3%.

### Emended description of* Tundrisphaera lichenicola* Kulichevskaya et al. 2017

Strain characteristics are as described before^22^ with the following modifications: The type strain has a genome size of 7.80 Mbp (chromosome and two plasmids) and a DNA G + C content of 64.7%.

### Emended description of the genus *Tundrisphaera*

Characteristics of the genus are as previously described^22^ with the following modifications: Chemoheterotrophic aerobes, either strictly aerobic or capable of growth under microaerobic conditions. Neutrophilic or moderately acidophilic. High DNA G + C content from 65 to 70%.

## Data Availability

The 16S rRNA gene and genome sequences of strain TA3^T^ and *T. lichenicola* P12^T^ are available from the NCBI GenBank database under the accession numbers provided in the Material and methods section.
